# Supplementary material for: Identification the Cross-Reactive or Species-Specific Allergens of Tyrophagus putrescentiae and Development Molecular Diagnostic Kits for Allergic Diseases
Source: Diagnostics (Basel). 2020 Sep 2;10(9):665. doi: 10.3390/diagnostics10090665 (PMC7555383; doi:10.3390/diagnostics10090665)
Supplement: Supplementary file 1 [file diagnostics-10-00665-s001.pdf]

Supplementary Table 1

Sequence comparison of allergens between *T. putrescentiae* and *D. pteronyssinus*

T. putrescentiae **allergens**	***D. pteronyssinus*** **allergens**	**Protein Identity**	**Protein Similarity**
**Tyr p 1**	**Der p 1**	27%	45%
**Tyr p 2**	**Der p 2**	36%	58%
**Tyr p 3**	**Der p 3**	40%	56%
**Tyr p 7**	**Der p 7**	28%	48%
**Tyr p 8**	**Der p 8**	66%	80%
**Tyr p 10**	**Der p 10**	80%	90%
**Tyr p 20**	**Der p 20**	85%	92%

Analyzed by Basic Local Alignment Search Tool (BLAST) in the Website of National Center for Biotechnology information (NCBI)(https://blast.ncbi.nlm.nih.gov/Blast.cgi)
